# What if children with psychiatric problems disagree with their clinicians on the need for care? Factors explaining discordance and clinical directions

**DOI:** 10.1186/s13034-022-00448-z

**Published:** 2022-02-14

**Authors:** Richard Vijverberg, Robert Ferdinand, Aartjan Beekman, Berno van Meijel

**Affiliations:** 1 Department of Child and Adolescent Psychiatry, GGZ Delfland Psychiatric Institute, Delft, The Netherlands; 2 Department of Health, Inholland University of Applied Sciences, Sports & Social Work, Amsterdam, The Netherlands; 3 Department of Psychiatry, Amsterdam UMC (VUmc), Amsterdam Public Health Research Institute, Amsterdam, The Netherlands; 4 Parnassia Psychiatric Institute, The Hague, The Netherlands

**Keywords:** Childhood, Clinicians, Disagreement, Concordance, Care Needs, Mental health care

## Abstract

**Background:**

Children and adolescents in mental healthcare often perceive their care needs and necessary treatment differently from their clinicians. As such discordance between young patients and clinicians may obstruct treatment adherence and compromise treatment outcomes, it is important to understand the factors associated with it. We therefore investigated the factors associated with patient–clinician discordance with regard to care needs in various areas of functioning.

**Methods:**

A cross-sectional study involving 244 children/adolescents aged 6–18 participating with their clinicians in treatment at a specialized mental healthcare center. As a previous study conducted by our research group had found the greatest patient–clinician discordance in three CANSAS care needs—“mental health problems,” “information regarding diagnosis and/or treatment,” and “making and/or keeping friends”—we used univariable and multivariable statistics to investigate the factors associated with discordance regarding these three care needs.

**Results:**

patient–clinician discordance on the three CANSAS items was associated with child, parent, and family/social-context factors. Three variables were significant in each of the three final multivariable models: dangerous behavior towards self (child level); severity of psychiatric problems of the parent (parent level); and growing up in a single-parent household (family/social-context level).

**Conclusions:**

To deliver treatment most effectively and to prevent drop-out, it is important during diagnostic assessment and treatment planning to address the patient’s care needs at all three levels: child, parent and family/social context.

## Background

If patients and clinicians in mental healthcare are to collaborate effectively, it is crucial that they agree on the care needs that need to be addressed [1–4]. By facilitating shared decision-making on treatment goals and interventions [5, 6], such agreement opens the gates to treatment adherence and effective treatment [7, 8].

Care needs can be defined as physical, psychological, social or environmental calls for aid, care or service in solving a problem [9]. These needs can either be ‘met’, which implies that a patient is receiving appropriate care, or ‘unmet’, which means that they are not being addressed adequately [10].

Children and adolescents who receive specialized mental healthcare often disagree with clinicians about unmet care needs [11–13]. In a previous study, we used the Camberwell Assessment of Need Short Appraisal Schedule.

(CANSAS) to examine the extent to which children and adolescents agreed or disagreed with their clinicians on a broad range of care needs, such as physical needs (e.g. do you have a physical disability or physical illness for which you have a need for care/help?), psychological needs (e.g. do you have any mental health problems for which you have a need for care/help?), social needs (e.g. do you have problems with making and/or keeping friends for which you have a need for care/help?), and environmental needs (e.g. do you have problems with getting access to or/and using modern communication tools for which you have a need for care/help?) [13]. We found that children and adolescents generally reported fewer unmet care needs than their clinicians. The highest discordance was found on the CANSAS items “mental health problems,” “information regarding diagnosis and/or treatment,” and “making and/or keeping friends”.

It is likely that treatment outcomes are influenced by discordance in these clinically relevant areas [1, 3]. Thus, as the presence of mental health problems is the primary reason for providing treatment, a lack of agreement between patient and clinician on this presence would deprive treatment of its fundamental reason for being: the lack of a fundamental basis for treatment [5]. Similarly, with respect to “information regarding diagnosis and/or treatment,” if a clinician believes that a patient might benefit from information about the diagnosis and options for treatment, but the patient does not see it as important, it is possible that the patient will have little interest in the treatment approach that the clinician proposes to deliver [14]. Discordance on the need to strengthen the patient’s social network shows that a clinician has observed that a patient is not functioning well in her/his social environment, but that the patient does not consider this to be a problem [14, 15].

Such discordance on care needs can undermine effective collaboration, and may also reduce treatment effects [16]. It is important to clinical practice to improve our understanding of this discordance [17], and also of the factors related to it [18]. As these factors had not yet been identified, this study was intended to fill the gap by focusing explicitly on the three CANSAS items identified above.

To categorize candidate predictors of discordance, we used the Bronfenbrenner model [19], which describes factors that influence a child’s functioning at three levels: that of (i) the child itself, (ii) that of his or her parents, and (iii) that of his or her family and social context. Given the lack of empirical research on discordance between children/adolescents and their clinicians regarding unmet care needs, we used studies that focused on factors associated with psychiatric problems and agreement on them.

With regard to the child level, existing studies suggest that children with more severe psychiatric problems in general, or with more severe internalizing and externalizing problems in particular, reported fewer mental health problems than their clinicians did [1, 3, 20, 21]. It has also been reported that dangerous behaviors towards oneself, rule-breaking behavior, and a higher age are associated with lower patient–clinician concordance on the presence of mental health problems [1, 3, 20].

With regard to the parent level, the literature shows that parents with higher levels of stress and more severe psychiatric problems report more mental health problems in their children than their children do [1, 21–23].

With regard to the level of the family/social context, the literature suggests that clinicians report more mental health problems than children or adolescents do in cases that involve lower family socio-economic status (SES), those that involve growing up in single-parent households, those that involve more problems with peers, and those that involve more problems at school [1, 24–29]. Discordance between patients and clinicians with regard to the severity of psychiatric problems was also predicted by greater child-parent discordance on the presence of mental health problems, and lower quality of the parent- child relationship [1, 3, 20, 30–32]. Given this review of literature, our a priori hypothesis was that discordance between clinicians and children/adolescents is predicted by predictors at all three levels, i.e., child, parent, and family/social context.

## Methods

### Design

Factors associated with patient–clinician discordance regarding unmet care needs were investigated using a cross-sectional design.

### Setting

The study was conducted at the department of child and adolescent psychiatry at a large specialized mental healthcare institution in the Netherlands. This department had two general outpatient clinics and one youth-Assertive Community Treatment team (ACT). Diagnostic assessments and treatment (e.g., cognitive behavioral therapy, family support, and pharmacological treatment) were provided by three child psychiatrists, seventeen psychologists, five clinical nurse specialists, and two mental health nurses.

### Participants

The target population consisted of all 6 to 18-year-olds who had been referred to the, and their clinicians. Only one child per household was included in the study. A total of 467 patients were eligible for inclusion.

**Fig. 1:**
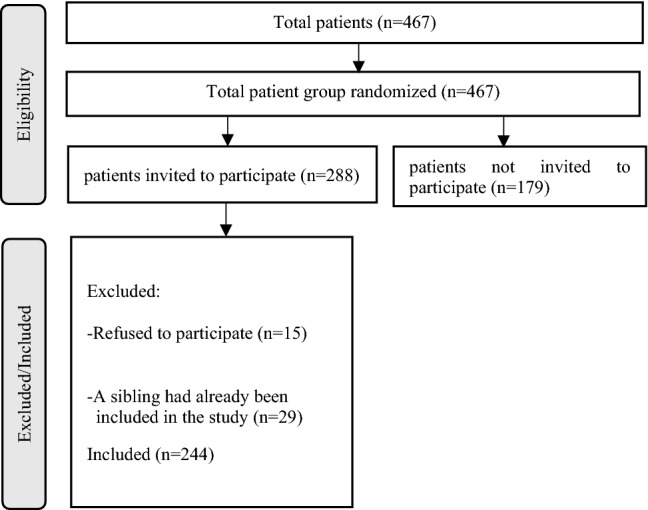
Participant flow diagram

We invited all patients referred for ACT during the inclusion period to participate in the study. Also, patients referred to a general outpatient center belonged to the target group of this study. To include an (approximately) equal number of ACT and outpatient patients, each month, the first six patients referred to one of the outpatient centers in that particular month, were included in the study. Some patients refused to participate (n = 15), or were excluded because they had a sibling who participated in the study (n = 29). The final sample consisted of 244 patients. Figure 1 shows the flow chart of the inclusion process.

### Ethical approval

The study was approved by the following: the Medical Ethical Committee at VU University Medical Center Amsterdam (protocol no. 2015.245); the Scientific Committee at the Amsterdam Public Health Research Institute; and the local research committee at the participating mental health institution.

Separately, participating children/adolescents and clinicians received written and oral information on the research project. In accordance with prevailing Dutch legislation, the written consent of parents and/or children/adolescents was obtained on the following basis: (i) if children were younger than 12, only parents were asked for consent; (ii) if children were aged between 12 and 16, parents and children were both asked for consent; and (iii) if adolescents were 16 or older, informed consent was obtained only from the adolescents themselves.

### Measurement instruments

#### Sample descriptives

The Demographic Information Questionnaire (DEMOG) child version was used to establish age, gender, and country of birth [33]. The Neuropsychiatric Interview for Children and Adolescent (MINI-KID) was used to establish the patients’ psychiatric diagnoses [34]. The MINI-KID generates reliable and valid psychiatric diagnoses for children and adolescents (AUC = 0.94, sensitivity 0.61–1.00, specificity 0.81–1.00) [34]. If a child was aged 12 or above, the MINI-KID was administered to the child alone. If a child was younger, the MINI-KID was administered in the presence of one of the parents. Parents were allowed to clarify questions for their child. For disorders that were not covered by the MINI-KID (personality disorders, autism spectrum disorders), clinical diagnoses were used.

### Assessment of care needs

To assess a patient’s unmet care needs, we used the Camberwell Assessment of Need Short Appraisal Schedule (CANSAS) [35], which covers 25 care need items that can be scored on a three-point scale. The response format is 0 = no need, 1 = met need, and 2 = unmet need. Cronbach’s alpha of the total CANSAS-score of the children/adolescents and their clinicians was good (0.86 and 0.89, respectively) in the present study, and even somewhat higher than the alpha of 0.78 in previous research [36].

The CANSAS was used in the form of an interview. If a child was aged 12 or older, it was administered to the child alone. If the child was younger, it was administered in the presence of one of the parents. At the start of the interview, parents were instructed not to answer for the child, but to clarify the questions in such a way that the child was able to answer the question from her or his own perspective. Simultaneously, the clinician also completed the CANSAS scoring form on the basis of all the clinical information available.

### Outcomes

Three dependent variables were studied: discordance between young people and their clinicians for (i) unmet care needs regarding mental health problems, (ii) unmet care needs regarding information on diagnosis and treatment, and (iii) unmet care needs regarding making and/or keeping friends.

To determine the presence or absence of patient–clinician discordance, scores for each of the three CANSAS items were recoded into 0 = no need/met need, and 1 = unmet need. Next, the item score of the clinician was subtracted from the patient’s score: 0 = concordance, and 1 or − 1 = discordance. As an explorative investigation, the present study did not focus on the nature of discordance i.e., on whether the clinician reported more care needs than the patient, or vice versa. Hence, all negative scores (= − 1) were recoded into positive ones (= 1).

### Predictors

#### Child factors

Candidate predictors at the child level were assessed as follows:

- *Severity of psychiatric problems* The Strength and Difficulty Questionnaire (SDQ, parent version) was used to assess the severity of mental health problems of the child from the parent’s perspective [37]. SDQ is a questionnaire that scores 33 items on a 3-point scale, in which 0 = not true, 1 = somewhat true, and 2 = certainly true [38]. Internal consistency of “total difficulties score” was acceptable in our study (0.75), and similar as in previous research [39].
- *Severity of internalizing problems and externalizing problems* To measure severity of internalizing and externalizing problems, we used two SDQ (parent version) subscales: “internalizing problems” and “externalizing problems” [36]. Cronbach’s alpha’s of these scales were acceptable in our sample (0.73 and 0.78 respectively), and somewhat higher than in previous research (0.66 and 0.62 respectively) [39].
- *Dangerous behavior towards self* To measure whether a patient currently showed dangerous behavior towards themselves, we used the MINI-KID domain “suicidal risk” (no = 0, or yes = 1) [34].
- *Rule-breaking behavior* The MINI-KID domains “conduct disorder” and “oppositional deviant disorder” were used to estimate rule-breaking behavior (diagnosis absent = 0, diagnosis present = 1) [34].
- *Age* The age of the child/adolescent was measured using the DEMOG [33].

### Parent factors

Candidate predictors at the parent level were assessed as follows:

- *Degree of parental stress* The Parental Stress Scale was used to measure the degree of parental stress by asking primary caregivers the following question: How much stress do you experience as a result of parenting (on a scale ranging from 1 to 10)?
- *Severity of psychiatric problems* The Health of the Nation Outcomes Scale (HoNOS) sum score was used to measure the severity of the parent’s psychiatric problems [40]. The HoNOS consists of 12 items to be scored on a 5-point-Likert scale, ranging from 0 (no problems) to 4 (severe problems). Internal consistency of “total score” was good in the present study (0.84), and somewhat higher than in previous research (0.78) [41].

### Family/social-context factors

Candidate predictors at the family/social-context level were assessed as follows:

- *Family SES *Family SES, expressed as the highest educational achieved by the parents, was measured using the DEMOG-Adult.
- *Growing up in a single-parent household *The DEMOG-Adult was also used to determine whether a child was growing up in single-parent or two-parent household [33].
- *Severity of problems with peers *The Kidscreen-27 (parent version) “friends” subscale was used to assess problems with peers as perceived by the parents [42]. This subscale comprises spending time with friends, fun with friends, support from friends, the extent to which a child could trust his/her friends. Internal consistency of the subscale “friends” was excellent in the present study (0.92), and higher than in previous research (0.78) [43]. Originally, higher item scores on Kidscreen-27 reflect better functioning, and range from 0 (= never) to 4 (= always). As we wanted to use an indicator that reflected greater severities of problems with friends as a candidate predictor, we recoded all item scores (0 = 4, 1 = 3, 2 = 2, 3 = 1, 4 = 0) before calculating a sum score.
- *Severity of problems related to school* To measure parents’ view of the severity of their child’s school problems, we used the parent version of the Kidscreen-27 subscale “school and learning,” which taps “had a good time at school,” “it went well at school,” “was able to pay attention in class,” and “quality of contact with teachers” [42]. A sum score was for this scale was calculated similarly as for the scale regarding “problems with peers.” The subscale”school and learning” showed good internal consistency (Cronbach’s alpha = 0.86), which was higher than in previous research (0.78) [43].
- *Severity of child-parent discordance on mental health problems* The child and parent version of the Strength and Difficulty Questionnaire (SDQ) were used to assess the severity of child-parent discordance regarding the presence of mental health problems [37]. Higher item scores of the SQD reflect more difficulties, and range from 0 (= not true) to 2 (= certainly true). The discordance was calculated by first subtracting the parent score from the child score for each item separately, which yielded discrepancy scores for each item. We then recoded all negative scores as positive scores. Finally, we summed all discrepancy scores [38]. A higher sum score thus indicates greater discordance.
- *Quality of the parent–child relationship* The “parent version” of the Kidscreen-27 “family” subscale was used to assess the quality of the parent–child relationship [42]. The “family” subscale covers 3 items: “support from parents,” “treated fairly by parents,” and “communication with parents.” Items are scored on a 5-point-Likert scale ranging from 0 (= never) to 4 (= always). To calculate the quality of the parent–child relationship, the scores of all 3 items were summed [42]. Internal consistency of “family” was acceptable (Cronbach’s alpha = 0.72), though somewhat lower than in previous research (0.78) [43].

### Data analysis

To analyze background characteristics, we first calculated descriptive statistics of the sample. Next, we conducted a set of univariable binary logistic regression analyses by using (i) concordance/discordance between young patients and their clinicians for each of the three outcomes variables (“mental health problems,” “information regarding diagnosis and/or treatment,” and “making and/or keeping friends”); and (ii) candidate predictors at all three levels of the Bronfenbrenner model (child / parent / family, social context). A separate regression analysis was performed for each candidate predictor (*P* < 0.05) [44], and yielded information on predictors at the child, parent, and family/social-context levels that predicted discordance between patient and clinician regarding the three outcomes. Our a priori hypothesis was that discordance between clinicians and children/adolescents would be predicted by predictors at child level, parent level, and family/social-context level. Since predictors at different levels may correlate despite being significant in univariable analyses, we then conducted stepwise multivariable logistic regression analyses to identify predictors at each level that were independent of other predictors, either at the same level, or at other levels. Therefore, stepwise multivariable logistic regression analyses were conducted for each of the three outcomes variables. As a first step, all child-level predictors that were significant in the set of univariable analyses were entered as possible predictors. Next, variables at parent level were entered, following by variables at family/social-context level. This step-by-step approach did not violate the statistical rule of 10 events per 1 variable [45, 46]. To test the assumptions of linearity and homoscedasticity, we generated a scatter plot of the standardized residuals [47], and tested assumptions of the logistic regression analyses for indications of multicollinearity by investigating the variance inflation factor (VIF) [44]. To measure the predictive value of models, we used the Hosmer and Lemeshow goodness-of-fit-test. Nagelkerke R^2^ was used to obtain an indication of the strength of the relationship between the predictor and the outcome variable [44]. All statistical analyses were performed using SPSS version 24.

## Results

### Sample characteristics

Table 1 shows the characteristics of the patients in the study sample (n = 244). Mean age was 12.4 years (sd = 3.3). A majority of the patients were boys (57.2%). Most patients were growing-up in a two-parent household (66.3%). The most frequent diagnoses were attention deficit hyperactivity disorder (43.4%), anxiety disorder (36.5%), autism spectrum disorder (25.4%), mood disorder (21.7%), and behavior disorder (20.9%).

**Table 1:** Sample characteristics of the child or adolescent who received treatment

	Patient	
	N = 244	
Age (sd)	Total mean	12.4 (3.3)
	range	6–18
	Boys mean	11.8 (3.2)
	range	6–17
	Girls mean	13.1 (3.2)
	range	6–18
Gender	Boys	57.2%
	Girls	42.8%
Country of birth	The Netherlands	95.9%
	Other	4.1%
Clinical diagnoses	ADHD	43.4%
	Anxiety	36.5%
	ASD	25.4%
	Mood	21.7%
	Behavior	20.9%
	Somatoform	6.6%
	Personality	2.9%
	Psychotic	2.0%
	Drugs/alcohol	1.6%
	Other	2.0%
GAF-score (sd)	Mean	50.5 (8.2)
	Range	15–75
Living situation	Two parent	66.3%
	Single parent	33.7%

N: number of included patients; sd: standard deviation; ADHD: attention deficit hyperactivity disorder; ASD: autism spectrum disorder; GAF: general assessment of functioning

### Mental health problems

The univariable analyses presented in Table 2 show that discordance between patients and clinicians on unmet mental healthcare needs was associated with all but three candidate predictors: (i) severity of the child’s externalizing psychiatric problems, (ii) family SES, and (iii) quality of the parent–child relationship.

**Table 2:** Factors associated with patient–clinician discordance on unmet care needs with regard to:

	Mental health problems				Information regarding diagnosis and/or treatment				Making and keeping friends			
	N	OR	95% CI	*P*	N	OR	95% CI	*P*	N	OR	95% CI	*P*
*Child level*												
Severity of psychiatric problems	244	1.077	1.034–1.123	** < 0.001**	244	1.086	1.042–1.131	** < .001**	244	1.113	1.065–1.164	** < .001**
Severity of internalizing problems	244	1.129	1.033–1.235	**0.008**	244	1.070	0.987–1.159	0.099	244	1.152	1.051–1.263	**0.003**
Severity of externalizing problems	244	1.094	0.975–1.229	0.126	244	1.182	1.046–1.337	**0.007**	244	1.152	1.022–1.300	**0.021**
Dangerous behavior												
Dangerous behavior towards self (yes/no)	244	5.268	2.980–9.312	** < 0.001**	244	4.182	2.404–7.277	** < 0.001**	244	5.582	3.156–9.871	** < 0.001**
Rule-breaking behavior (yes/no)	244	3.647	2.024–6.571	** < 0.001**	244	3.072	1.760–5.364	** < 0.001**	244	3.632	2.031–6.494	** < 0.001**
Age *4–11* *12–18*	243 90 153	1.823	1.033–3.219	**0.038**	243 90 153	1.332	0.776–2.288	0.299	243 90 153	1.978	1.122–3.486	**0.018**
*Parent level*												
Degree of parental stress	244	1.525	1.316–1.768	** < 0.001**	244	1.265	1.121–1.427	** < 0.001**	244	1.407	1.229–1.609	** < 0.001**
Severity of parent’s psychiatric problems	244	1.232	1.163–1.305	** < 0.001**	244	1.163	1.107–1.221	** < 0.001**	244	1.236	1.166–1.310	** < 0.001**
*Family/social-context level*												
Family’s socioeconomic status *Basic* *Intermediate* *High*	244 86 112 46	0.835 0.703	0.467–1.494 0.327–1.508	0.644 0.544 0.365	244 86 112 46	0.553 0.739	0.310–0.984 0.357–1.531	0.131 0.044 0.416	244 86 112 46	0.892 0.602	0.502–1.587 0.278–1.306	0.433 0.698 0.199
Child growing up in a single-parent household	244	4.588	2.618–8.042	** < 0.001**	244	3.110	1.813–5.335	** < 0.001**	244	3.510	2.030–6.072	** < 0.001**
Quality of the parent–child relationship	238	0.997	0.935–1.063	0.920	238	0.996	0.935–1.061	0.904	238	0.975	0.915–1.039	0.435
Degree of discordance between parent and child on the child’s mental health problems	190	1.168	1.096–1.245	** < 0.001**	190	1.168	1.096–1.245	** < 0.001**	190	1.166	1.094–1.242	** < 0.001**
Severity of problems with peers	244	1.125	1.055–1.199	** < 0.001**	244	1.098	1.031–1.168	**0.004**	244	1.120	1.050–1.193	**0.001**
Severity of problems related to school	244	1.078	1.009–1.152	**0.026**	244	1.067	1.000–1.139	**0.050**	244	1.079	1.010–1.152	**0.024**

Univariable analysis: individual binary logistic regression analyses of each candidate factor performed separately; N: Number of patients; OR: Odds ratio; CI: Confidence interval; *P*-value < 0.05 is considered statistically significant (in bold)

Table 3 shows that the final step of the multivariable analysis produced two significant predictors at child level: dangerous behavior towards self, and rule-breaking behavior. Significant parent-level predictors were degree of parental stress, and severity of the parent’s psychiatric problems. Predictors at family/social-context level were growing up in a single-parent household, and degree of discordance between parent and child on the severity of the child’s mental health problems. The final model showed a good fit of the data (Hosmer–Lemeshow, P = 0.645), and a moderately strong relationship between predictor variables and outcome (Nagelkerke R^2^ = 0.575).

**Table 3:** Multivariable analyses of factors associated with patient–clinician discordance on unmet care needs with regard to mental health problems

	Child multivariable model				Child and parent multivariable model				Child, parent and family/social context multivariable model			
	N	OR	95% CI	*P*	N	OR	95% CI	*P*	N	OR	95% CI	*P*
*Child level*												
Severity of psychiatric problems	243				243				186			
Severity of internalizing problems		1.072	1.011–1.137	**0.020**		0.992	0.938–1.048	0.769				
Dangerous behavior		1.041	0.905–1.199	0.572								
Dangerous behavior towards self (yes/no)		4.187	2.187–8.015	** < 0.001**		3.813	1.918-.7.579	** < 0.001**		3.716	1.558–8.867	**0.003**
Rule-breaking behavior (yes/no)		2.927	1.517–5.647	**0.001**		2.398	1.151–4.995	**0.019**		3.086	1.273–7.481	**0.013**
Age *4–11* *12–18*	243 90 153	1.304	0.662–2.567	0.443								
*Parent level*												
Degree of parental stress						1.311	1.110–1.549	**0.001**		1.233	1.021–1.490	**0.030**
Severity of parent’s psychiatric problems						1.180	1.104–1.261	**0.001**		1.137	1.054–1.227	**0.001**
*Family/social context level*												
Child growing up in a single-parent household										3.022	1.300–7.025	**0.010**
Degree of discordance between parent and child on the child’s mental health problems										1.082	0.997–1.174	0.058
Severity of problems with peers										1.083	0.885–1.196	0.117
Problems related to school												
Severity of problems related to school										1.013	0.908–1.130	0.816

Multivariable analysis: binary regression analyses of the predictors that were significant in the univariable analysis, performed simultaneously; n: number of patients; CI: Confidence interval; P-value < 0.05 is considered statistically significant (in bold)

Child-level: Omnibus test, Step P = 0.000, Model P =  < 0.000, Hosmer–Lemeshow, P = 0.058, Nagelkerke R2 = 0.295

Child-parent-level: Omnibus test, Step P = 0.000, Model P =  < 0.000, Hosmer–Lemeshow, P = 0.437, Nagelkerke R2 = 0.499

Child-parent-family/social-context level: Omnibus test, Step P = 0.000, Model P =  < 0.000, Hosmer–Lemeshow, P = 0.645, Nagelkerke R2 = 0.575

### Information on diagnosis and treatment

With regard to the univariable analysis, Table 2 shows that discordance regarding the need for information on diagnosis and treatment was significantly associated with all predictor variables, except for severity of internalizing problems, family SES, higher age of the child, and quality of the parent–child relationship.

With regard to the final step of the multivariable analyses, Table 4 shows that, at the child level, discordance on the care need “information on diagnosis and treatment” was predicted by dangerous behavior towards self. At the parent level, discordance was predicted by the presence of the parent’s psychiatric problems. At family/social-context level there were two significant predictors: growing up in a single-parent household, and discordance between parent and child on the presence of mental health problems in the child. The final model fitted the data well (Hosmer–Lemeshow, P = 0.571), and showed a moderately strong relationship between predictor variables and outcome (Nagelkerke R^2^ = 0.451).

**Table 4:** Multivariable analyses of factors associated with patient–clinician discordance on unmet care needs with regard to information regarding diagnosis and/or treatment

	Child multivariable model				Child and parent multivariable model				Child, parent and family/social context multivariable model			
	N	OR	95% CI	*P*	N	OR	95% CI	*P*	N	OR	95% CI	*P*
*Child level*	243				243				186			
Severity of psychiatric problems		1.060	1.010–1.112	**0.018**		1.034	0.985–1.085	0.179				
Severity of externalizing problems		1.120	0.971–1291	0.120								
Dangerous behavior towards self (yes/no)		4.194	2.295–7.664	** < 0.001**		2.997	1.621-.5.540	** < 0.001**		3.334	1.478–7.517	**0.004**
Rule-breaking behavior (yes/no)		2.288	1.249–4.192	**0.007**		2.002	1.063–3.769	**0.032**		1.920	0.858–4.300	0.113
*Parent level*												
Degree of parental stress						1.069	0.929–1.229	0.352				
Severity of parent’s psychiatric problems						1.107	1.047–1.170	** < 0.001**		1.125	1.054–1.200	** < 0.001**
*Family/social context level*												
Child growing up in a single-parent household										2.336	1.076–5.072	**0.032**
Degree of discordance between parent and child on the severity of the child’s mental health problems										1.085	0.964–1.151	**0.037**
Severity of problems with peers										1.053	0.975–1.070	0.155
Problems related to school												
Severity of problems related to school										1.015	0.926–1.113	0.746

Multivariable: binary regression analyses of the predictors that were significant in the univariable analysis, performed simultaneously; n: number of patients; CI: Confidence interval; *P*-value < 0.05 is considered statistically significant (in bold)

Child-level: Omnibus test, Step P = 0.000, Model P =  < 0.000, Hosmer–Lemeshow, P = 0.355, Nagelkerke R^2^ = 0.261

Child-parent-level: Omnibus test, Step P = 0.000, Model P =  < 0.000, Hosmer–Lemeshow, P = 0.917, Nagelkerke R^2^ = 0.329

Child-parent-family/social-context-level: Omnibus test, Step P = 0.000, Model P =  < 0.000, Hosmer–Lemeshow, P = 0.571, Nagelkerke R^2^ = 0.451

### Making and keeping friends

Table 2 shows that discordance on the care need “making and keeping friends” was significantly associated with all variables in the univariable analyses, except for family SES, and quality of the parent–child relationship.

With regard to the final step of the multivariable analyses, Table 5 showed one child-level predictor: dangerous behavior towards self. Parent-level predictor was severity of the parent’s psychiatric problems. At the family/social-context level, discordance was predicted by growing up in a single-parent household. The model showed a good fit of the data (Hosmer–Lemeshow, P = 0.514), and a moderately strong relationship between the predictor variables and the outcome (Nagelkerke R^2^ = 0.527).

**Table 5:** Factors associated with patient–clinician discordance on unmet care needs with regard to making and keeping friends

	Child multivariable model				Child and parent multivariable model				Child, parent and family/social context multivariable model			
	N	OR	95% CI	*P*	N	OR	95% CI	*P*	N	OR	95% CI	*P*
*Child level*												
Severity of psychiatric problems	243				243				181			
Severity of internalizing problems		1.114	1.049–1.247	**0.002**		1.049	0.993–1.109	0.088				
Severity of externalizing problems		0.963	0.823–1.128	0.644								
Dangerous behavior		0.996	0.790–1259	0.975								
Dangerous behavior towards self (yes/no)		4.955	2.493–9.849	** < 0.001**		4.458	2.239-.8.875	** < 0.001**		4.320	1.867–9.996	**0.001**
Rule-breaking behavior (yes/no)		2.709	1.389–5.281	**0.003**		2.286	1.110–4.710	**0.025**		1.683	0.725–3.903	0.226
Age *4–11* *12–18*	243 90 153	1.642	0.814–3.314	0.166								
*Parent level*												
Degree of parental stress						1.162	0.991–1.363	0.065				
Psychiatric problems												
Severity of parent’s psychiatric problems						1.166	1.093–1.244	** < 0.001**		1.166	1.087–1.251	** < 0.001**
*Family/social-context level*												
Child growing up in a single-parent household										2.410	1.059–5.487	**0.036**
Degree of discordance between parent and child on the severity of the child’s mental health problems										1.081	1.000–1.169	0.051
Severity of problems with peers										0.921	0.838-.1.013	0.089
Severity of problems related to school										1.000	0.900–1.112	1.000

Multivariable: binary regression analyses of the predictors that were significant in the univariable analysis, performed simultaneously; n: number of patients; CI: Confidence interval; *P*-value < 0.05 is considered statistically significant (in bold)

Child-level: Omnibus test, Step P = 0.000, Model P =  < 0.000, Hosmer–Lemeshow, P = 0.657, Nagelkerke R^2^ = 0.368

Child-parent-level: Omnibus test, Step P = 0.000, Model P =  < 0.000, Hosmer–Lemeshow, P = 0.969, Nagelkerke R^2^ = 0.496

Child-parent-family/social-context-level: Omnibus test, Step P = 0.000, Model P =  < 0.000, Hosmer–Lemeshow, P = 0.514, Nagelkerke R^2^ = 0.527

### All three care needs

patient–clinician discordance on all three predefined CANSAS items was associated with child, parent, and family/social-context factors. The three final multivariable models found three common significant predictors: dangerous behavior towards self (child level); severity of the parent’s psychiatric problems (parent level); and growing up in a single-parent household (family/social-context level).

### Association between gender and variables

We tested for associations between gender and variables included in our models, however these associations were absent.

## Discussion

This study examined associations between patient, parent, and family/social-context variables on the one hand, and patient–clinician discordance on regarding unmet need for care on the other. We investigated discordance for the following unmet needs for care: (i) mental health problems, (ii) information regarding diagnosis and/or treatment, and (iii) making and/or keeping friends. In the present sample, discordance on these three unmet care needs indicated mainly that clinicians deemed care to be necessary, whereas patients did not. As we had hypothesized, discordance between clinicians and children/adolescents was predicted by predictors at child, parent, and family/social-context levels.

Most of the variables that were analyzed univariably were associated with discordance between patient and clinician on all three care needs (see Table 2). As stated above, we conducted multivariable stepwise logistic regression analyses to identify which predictors predicted this discordance independently of other predictors, and to investigate whether variables at all three levels were needed to obtain the strongest predictive model. As this resulted in three final models (for all three outcomes) that encompassed predictors from all levels, discordance between patients and clinicians was truly predicted by information on children, parents, and the family/social context.

Below, we summarize which variables predicted discordance with respect to the three care needs in the multivariable models (see Tables 3, 4 and 5). As these analyses showed which predictors were the most useful in predicting discordance, the final statistical models tended to present a prototypical picture of patients who disagreed with their clinician on their need for care, and of their parents and family/social context.

### Disagreement on all three outcomes predicted by child level variables (final set of analyses)

Many patients who disagree with their clinicians on the three care needs we examined often show dangerous behavior towards themselves (e.g., suicide attempt, self-harming behavior). It seems that dangerous behavior is less a reason to seek help than a more modest demand for mental health support. This can be explained in various ways. Suicidal thoughts or self-harm may indicate that a patient no longer sees a way out, and thus tends to think that care will not be helpful [48]. Similarly, patients who harm themselves may consider self-harm to be a better way of coping with negative emotions than treatment is [48]. In many cases they may have lost confidence in the ability of other people to help relieve their suffering. Self-harm may lead to the immediate alleviation of negative thoughts or feelings (such as tension, anxiety or anger), or may increase social support or attention [49]. It is possible that such reinforcement affects a patient’s perceived need for care. In contrast, clinicians of patients who harm themselves may see opportunities for improvement, and may therefore indicate that care is needed.

### Disagreement on all three outcomes predicted by parent-level variables (final set of analyses)

Children and adolescents who tended to disagree with clinicians about all three care needs had parents with psychiatric problems. Due to these problems, clinicians may believe that these young people have more care needs, reasoning that the children of such parents are at greater risk, and thus require more attention. It is also the case that parents with psychiatric problems are more likely to report severer problems in their children, whatever the actual severity [50, 51]. This may lead clinicians to judge that care is needed, while the young people rate themselves as being less in need of help [1, 27]. Another possible explanation is that young people who grow up with parents with mental health problems have become accustomed to problems, and believe that they cannot be resolved [52, 53].

### Disagreement on all three outcomes predicted by family/social-context level variables (final set of analyses)

We found that many young people who disagreed with their clinicians on all three of the care needs studied had grown up in a single-parent household. It is conceivable that clinicians rate care needs more highly if they feel that a child is less protected, in view of the fact that there is one parent rather than two. Alternatively, parents who run a single-parent household may report relatively high problem levels, and stress the need for care, as they are caring for their children on their own. This may cause clinicians, too, to give higher ratings to need for care.

### Other significant findings

We found that the degree of a child’s rule-breaking behavior (as assessed after a standardized interview with the child), predicted patient–clinician discordance on unmet need for care for mental health problems.

It is possible that young patients who break rules are less aware of their problems, or do not see the need for change [54]. Any negative experiences with adults [55]—who make and enforce rules—may also negatively affect their motivation for collaborating with treatment intended to resolve problems [56].

For one of the three unmet care needs we investigated—unmet care needs regarding information regarding diagnosis and/or treatment—we found that the degree of discordance between parent and child also predicted discordance between patient and clinician. As proposed previously [13, 26, 51], this may mean that clinicians agree more with parents than with their children/adolescents, thus possibly indicating that clinicians take parents more seriously than they take young people.

Alternatively, children/adolescents who disagree with their parents may also tend to disagree with clinicians [50, 51]. Previous research showed that, irrespective of the actual severity, more parents with high stress levels tend to report a greater problem severity in their children [50, 51]. In theory, this might cause clinicians to give a higher rating to needs for care, and therefore to inflate patient–clinician disagreement. [1, 27]. However, we found that one specific association at least was independent of the degree of parental stress: that between child-parent disagreement and child/clinician disagreement.

### Clinical implications

In the context of personalized care, care needs should be assessed from the start of the treatment trajectory [57]. They can be addressed properly only if they are examined systematically from the perspectives of the key people involved in treatment [13, 57]. Our findings show that special attention should be paid to the particular perspective on care needs that applies to patients who harm themselves, exhibit suicidal behaviors, break rules, have parents with psychiatric problems, disagree with their parents on the presence of mental health problems, and grow up in a single-parent households. If patient–clinician perceptions differ, clinicians are advised to resolve the most important differences before treatment is delivered [58]. Given that positive treatment outcomes are associated with a good therapeutic relationship [4], it is important to debate differences in a way that enables the “bond” in the patient–clinician dyad to remain intact [16, 59]. However, due to the importance of factors at parent and family/context levels—such as parental psychiatric problems and growing up in a single-parent household—it is probably not effective to solve discordances solely through this dyad. It is therefore important to discuss patients' needs for care in the triad of patient, parent, and clinician, paying specific attention to the predictive factors outlined above that significantly contribute to discordance.

Fruitful therapeutic relationships within this triad will contribute to positive treatment outcomes and the prevention of drop-out from treatment. Establishing such relationships must start with a shared view of the care that is needed. It means that patients’ perspectives on care their needs should be taken seriously. It also means seeking shared goals and making decisions on interventions collaboratively—together *with* patients, not *for* them [56].

These guidelines for a care-needs-based approach provide a flexible framework that gives guidance to clinicians, while leaving them scope for appropriate action on individual and situational peculiarities. To encourage and facilitate discussion of different viewpoints on care needs, various patient-centered communication techniques can be used, including (i) motivational interviewing techniques that are characterized by bond-building, empathy, interpersonal sensitivity, and the provision of information [57]; and (ii) shared decision, which may help to establish a process for collaboratively making decisions about the care needs that will be targeted during treatment [58].

### Strengths/limitations

This study has several strengths. To our knowledge, it is the first to provide insight into factors that are associated with discordance between patients and clinicians on unmet care needs [26, 58, 59]. Due to our use of more than one outcome variable for child/adolescent-clinician discordance, we were able to identify factors that predict discordance on unmet care needs. Knowledge obtained may contribute to a more personalized form of care that enables patients to better identify with the treatment provided [10]. This knowledge may help patients to feel more engaged in treatment, and to prevent non-adherence and drop-out [60]. Finally, our use of hierarchical analyses made it possible to investigate relevant predictors at child, parent, and family/social-context levels.

A limitation is that our data were collected at a single mental health organization. For this reason, our results can be generalized only with reservations [61]. Further, although the present study identified a number of factors that may explain child–clinician discrepancies regarding need for care, it is important to realize that some potentially important factors were not studied. More specifically, it was neither investigated if ethnic or cultural differences between children and clinicians were associated with discrepancies, nor was it investigated if other, clinician-related factors, such as personality traits, gender, own experiences with the health care system, personal values, or stress levels, were associated with child-clinician discrepancies. The present study did not investigate whether ethnic or cultural background of the parents explained the association that was found between SES and mental health of the parents on the one hand, and child-clinician discrepancies on the other hand. Further research might clarify such issues. In future research, it might also be useful to include `attachment style´ as a candidate predicator, because there is some evidence that attachment style in adults is associated with the use of mental health services [62, 63].

## Conclusion

We found that discordance between young people and clinicians on unmet care needs were associated with factors at child, parent, and family/social-context levels. On this basis, we conclude that it is important to the effective delivery of treatment and the prevention of drop-out to address all three levels during diagnostic assessment and psychiatric treatment.

## Data Availability

The datasets used and/or analyzed during the current study are available from the corresponding author on reasonable request.

## Abbreviations

ASD: Autism spectrum disorder
ADHD: Attention deficit hyperactivity disorder
AUC: Area under the curve
CANSAS: Camberwell Assessment of Need Short Appraisal Schedule
CI: Confidence interval
GAF-score: Global Assessment of Functioning score
HoNOS: Health of the nation outcomes scale
MINI-KID: MINI International Neuropsychiatric Interview for Children and Adolescents
N: Number of patients
OR: Odds ratio
SD: Standard deviation
SDQ: Strength and difficulty questionnaire
SES: Social economic status
SPSS: Statistical Package for the Social Sciences
